# Epigenetics Regulates Reproductive Development in Plants

**DOI:** 10.3390/plants8120564

**Published:** 2019-12-02

**Authors:** Qiang Han, Arthur Bartels, Xi Cheng, Angela Meyer, Yong-Qiang Charles An, Tzung-Fu Hsieh, Wenyan Xiao

**Affiliations:** 1 Department of Biology, Saint Louis University, St. Louis, MO 63103, USAarthur.bartels@slu.edu (A.B.); xi.cheng@slu.edu (X.C.);; 2 Department of Biochemistry, Purdue University, West Lafayette, IN 47907, USA; 3 US Department of Agriculture, Agricultural Research Service, Midwest Area, Plant Genetics Research Unit, Donald Danforth Plant Science Center, MO 63132, USA; yong-qiang.an@ars.usda.gov; 4 Department of Plant and Microbial Biology, North Carolina State University, Raleigh, NC 27695, USA; thsieh3@ncsu.edu; 5 Plants for Human Health Institute, North Carolina State University, North Carolina Research Campus, Kannapolis, NC 28081, USA

**Keywords:** DNA methylation, dynamics, Polycomb group proteins, histone modifications, epigenetics, chromatin, siRNA, RdDM, gametogenesis, seed development

## Abstract

Seed, resulting from reproductive development, is the main nutrient source for human beings, and reproduction has been intensively studied through genetic, molecular, and epigenetic approaches. However, how different epigenetic pathways crosstalk and integrate to regulate seed development remains unknown. Here, we review the recent progress of epigenetic changes that affect chromatin structure, such as DNA methylation, polycomb group proteins, histone modifications, and small RNA pathways in regulating plant reproduction. In gametogenesis of flowering plants, epigenetics is dynamic between the companion cell and gametes. Cytosine DNA methylation occurs in CG, CHG, CHH contexts (H = A, C, or T) of genes and transposable elements, and undergoes dynamic changes during reproduction. Cytosine methylation in the CHH context increases significantly during embryogenesis, reaches the highest levels in mature embryos, and decreases as the seed germinates. Polycomb group proteins are important transcriptional regulators during seed development. Histone modifications and small RNA pathways add another layer of complexity in regulating seed development. In summary, multiple epigenetic pathways are pivotal in regulating seed development. It remains to be elucidated how these epigenetic pathways interplay to affect dynamic chromatin structure and control reproduction.

## 1. Introduction

Seed represents the major nutrient source for humans and domesticated animals and produces many important industrial polymers such as oils and starches. Seed is the product of a double fertilization event in angiosperms. Unlike mammals, plants feature a haploid gametophytic growth involving postmeiotic cell divisions before fertilization. In the flowering plants, a haploid megaspore after meiosis goes through three mitotic divisions to form an eight-nucleus seven-cell female gametophyte containing the egg, central cell, two synergids, and three antipodal cells. Two haploid nuclei fuse to make the diploid central cell nucleus in the female gametophyte [1,2]. In the male gametophyte, a haploid microspore after meiosis goes through a mitotic division to form one vegetative and one generative cell. The generative cell then divides mitotically into two haploid sperm cells. After a pollen grain settles on top of the pistil, the vegetative cell grows into a pollen tube and transports two sperms to ovules at the base of pistils. Double fertilization event occurs in the female gametophyte of the ovule. During fertilization, one sperm fertilizes the egg cell to generate a diploid embryo. The second sperm fertilizes the diploid central cell to generate a triploid endosperm, which supports embryo and seedling growth [1,2,3,4]. Embryogenesis in vascular plants generates a mature and functional embryo that can eventually grow into a whole plant after seed germination. During this process, primary plant tissues are differentiated and the basic body plan is formed [5]. The embryo goes through a series of stages morphologically characterized as globular, heart, torpedo, bent-cotyledon, and walking-stick stages in many plant species such as *Arabidopsis* [5,6]. After completing rapid cell divisions and differentiation, the young embryo shifts into a seed-filling and seed-maturation stage at which the seed volume expands and storage reserve is synthesized [7]. Seed maturation is an important process for seed quality and yield during late development.

DNA methylation usually refers to modification of cytosine to become 5-methylcytosine (5mC). Cytosine methylation regulates transposable element (TE) silencing, genomic imprinting, and gene transcription in mammals and plants. DNA methylation at CG dinucleotides is evolutionarily conserved in plants, mammals, and some fungi [8,9,10,11]. De novo DNA methyltransferase 3 (Dnmt3) initiates and DNA methyltransferase 1 (Dnmt1) maintains cytosine methylation in mammals [12,13]. Flowering plants have DNA methylation at CG, CHG, and CHH contexts (H = A, C, or T). In *Arabidopsis*, CG and CHG methylation is mainly maintained by DNA METHYLTRANSFERASE1 (MET1), an ortholog of Dnmt1 in mammals, and CHROMOMETHYLASE3 (CMT3), respectively [14,15,16,17,18,19]; CHH methylation is maintained by the de novo DNA methyltransferases DOMAINS REARRANGED METHYLTRANSFERASE1 and 2 (DRM1 and DRM2) and CMT2 [18,19,20,21,22]. DRM2 is also responsible for de novo methylation in all sequence contexts through the RNA-directed DNA methylation (RdDM) pathway mediated by RNA polymerase IV (Pol IV) that produces small interfering RNAs (siRNAs) [21,23].

Polycomb group (PcG) proteins were originally identified in *Drosophila* and are involved in silencing homeotic genes by maintaining the repressed chromatin state [24,25]. PcG proteins recognize and interact with polycomb response elements (PREs) that are scattered throughout the genome to silence expression of genes containing PREs and nearby genes in *Drosophila* [26]. Most PcG proteins form two major polycomb repressive complexes (PRC)—PRC1 and PRC2 [27]. PRC2 consists of four key components: enhancer of zeste (EZ), suppressor of zeste 12 (Suz12), extra sex combs (Esc), and the histone-binding protein p55 in *Drosophila*. Homologous components of PcG proteins have been identified and function in plant development, which will be reviewed later in this paper. The *Arabidopsis* homologs of PcG proteins in seed development were identified by genetic screening, and they include FERTILIZATION INDEPENDENT ENDOSPERM (FIE), FERTILIZATION INDEPENDENT SEED2 (FIS2), MEDEA (MEA), and MULTICOPY SUPPRESSOR OF IRA1 (MSI1) [28,29,30,31,32]. Mutations in these PcG genes result in endosperm proliferation in the absence of fertilization.

Histone modifications refer to post-translational covalent changes on histone tails that are essential for dynamic chromatin structure and many cellular processes [33,34,35,36]. To date, more than 100 different histone modifications have been identified, from intensively studied lysine methylation and acetylation, and serine and threonine phosphorylation to recently discovered modifications, such as crotonylation [37,38]. With advancement of recent technologies, such as chromatin immunoprecipitation with tiled microarray (ChIP chip) and next-generation sequencing, global patterns of histone modifications have been mapped in many organisms [39,40,41,42,43]. These research studies have shown that histone acetylation and methylation in genes are important for gene transcription. Specifically, histone modifications such as acetylation and methylation in gene-coding regions are involved in plant reproduction [39].

Imprinting refers to differential expression of alleles on the basis of their parent of origin, contributing to reproductive success in animals and plants [4,44,45,46]. Gene imprinting primarily happens in the plant endosperm [47,48], whereas it can occur in the embryo and other tissues throughout post-embryonic stages in mammals [49]. Imprinted genes affect endosperm growth and seed size in *Arabidopsis* and crop plants [50,51,52,53,54,55]. In addition, a few imprinted genes have been found to occur in the embryo in both *Arabidopsis* [56] and maize [47]. Mutations in some imprinted genes from one parental allele can cause seed abortion, whereas the mutation on the other parental allele has mostly no effect in plants [57]. On the other hand, mutations in many recently found imprinted genes have no obvious phenotypes, or functions of these genes in seed development remain to be discovered [58].

The RdDM pathway has been shown to be involved in imprinting in *Arabidopsis* [59]. Non-coding small RNAs (sRNAs) have important functions in growth and development in both mammals and plants [60]. sRNAs are generated from double-stranded RNAs through the activities of RNA DEPENDENT RNA POLYMERASE (RDR), DICER-LIKE (DCL), and ARGONAUTE (AGO) proteins [61,62]. The two main classes of sRNAs are siRNAs and microRNAs (miRNAs) that both negatively regulate gene expression by binding to complementary sequences of their target genes [61,63]. The 24 nt siRNAs can trigger DNA methylation through the RdDM pathway [60] and affect seed development [64]. Briefly, Pol IV transcribes non-coding single-stranded RNAs (ssRNAs) that will be synthesized to double-stranded RNAs (dsRNAs) by RDR2. Then, DCL3 cuts the dsRNAs [65] into 24 nt siRNAs, which are methylated at their 3′ end by HUA ENCHANCER1 (HEN1) and are loaded onto ARGONAUTE4 (AGO4) [66]. Pol V and KOW DOMAIN-CONTAINING TRANSCRIPTION FACTOR1 (KTF1) recruit AGO4 through the C-terminal domain of Pol V’s largest subunit, NUCLEAR RNA POLYMERASE E1 (NRPE1) [67,68,69]. In *Arabidopsis*, CLASSY (CLSY) 1–4, SUCROSE NONFERMENTING2 (SNF2)-related, and putative chromatin remodeling proteins are necessary for global DNA methylation [41]. The CLSY proteins regulate 24 nt siRNA generation and are important for Pol IV chromatin association [41]. Lastly, the siRNAs bound to AGO4 are thought to complementarily pair with the Pol V transcripts and recruit DRM2 to methylate at homologous genomic sites.

Recent evidence has shown that a non-canonical RDR6-dependent RdDM pathway exists in plants [70,71,72,73,74,75]. When a TE is transcribed by Pol II, the TE transcripts are converted into dsRNAs by RDR6. The dsRNAs are then processed into 21–22 nt siRNAs by DCL2 and DCL4. The 21–22 nt siRNAs are then loaded onto AGO1 and can subsequently target Pol V-scaffolding transcripts, resulting in de novo DNA methylation.

DNA methylation was first shown to be crucial for seed development by regulating proper expression of genes important for cell identity and hormone gradient establishment [76]. Since then, substantial progress has been made in epigenetic control of gametogenesis, embryogenesis, and seed maturation through epigenetic and epigenomic approaches. With rapid advancement of this field in recent years, it is challenging to summarize all results, along with sometimes seemingly contradictory results. The authors hope that this review summarizes the major progress during the last decade and stimulates discussion about future perspectives in epigenetic regulation of seed development.

## 2. Differential DNA Methylation in the Egg and Central Cell of the Female Gametophyte

**Figure 1:**
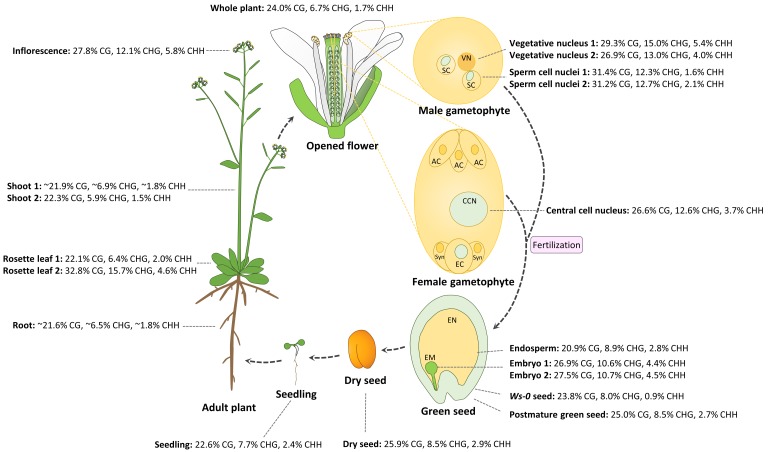
Dynamic DNA methylation patterns in the life cycle of *Arabidopsis*. DNA methylation percentage in each of CG, CHG, and CHH contexts represent the number of methylated cytosine sites relative to all sequenced cytosine sites in the same context. Abbreviations are as follows: vegetative nucleus (VN), sperm cell (SC), antipodal cell (AC), central cell nuclei (CCN), egg cell (EC), synergid cell (Syn), embryo (EM), endosperm (EN). Materials and references for methylation data are listed as follows: all materials were from Col-0 wild type except green seed from Ws-0. Data were from the following: whole plant [79]; shoot 1 and root [80]; shoot 2 [81]; rosette leaf 1 [82]; rosette leaf 2 [83]; inflorescence, sperm cell nuclei 1, vegetative nucleus 1, and embryo 2 [84]; sperm cell nuclei 2 and vegetative nucleus 2 [78]; central cell nucleus (24 h after stage 12 flower) and seedling (2 week old) [77]; endosperm and embryo 1 at mid-torpedo to early seed maturation stage (7–9 days after pollination (DAP)) [85]; Ws-0 green seed at globular stage, postmature green seed (Col-0), and dry seed (Col-0) [86].

The female gametophyte is formed from meiosis of a diploid megaspore mother cell followed by three mitotic divisions. The mature female gametophyte contains seven cells and eight nuclei including an egg cell and diploid central cell, which give rise to the embryo and endosperm after fertilization by the sperm cells, respectively. Until recently, little was known about DNA methylation profiles in a specific cell type in the female gametophyte due to difficulty in isolating abundant amount of genomic DNA from the specific cell type. By studying whole genome methylation in the central cell of *Arabidopsis* and rice, as well as the egg cell in rice, Park et al. found that DNA demethylation is initiated in the central cell in both *Arabidopsis* and rice, and demethylation requires DEMETER (DME), a DNA glycosylase in *Arabidopsis* [77]. In the rice female gametophyte, DNA methylation in the central cell is approximately 39.4% CG, 18.2% CHG, and 6.3% CHH, lower than 41.9% CG, 26.2% CHG, and 7.4% CHH in the egg cell, which is likely due to demethylation in the central cell [77]. In the central cell of *Arabidopsis*, global CG methylation levels (26.6%) are slightly lower than those in the sperm (31.3%), but higher than those in the endosperm (20.9%); CHG methylation levels (12.6%) are similar to those in the sperm (12.5%), and slightly higher than those in the endosperm (8.9%); CHH methylation levels (3.7%) are significantly higher than those in the sperm (1.6%–2.1%) and endosperm (2.8%) (Figure 1). Without considering variations in different experiments, CG, CHG, and CHH methylation in reproductive tissues and cells except the endosperm (inflorescence, vegetative, and sperm cells; central cells; and embryos) is higher than that in somatic vegetative tissues (shoots, rosette leaves, and seedlings, except the sample of “Rosette leaf 2”) in *Arabidopsis* (Figure 1; no egg cell methylome data available). Due to DME activity in the central cell, maternal hypomethylation of some TEs can cause small RNA expression in the endosperm, which might be moved to the embryo in order to methylate the TEs sharing homology or nearby genes via RdDM, thus fortifying the silencing of TEs in the embryo that passes its genetic information to the next generation [78].

**Figure 2:**
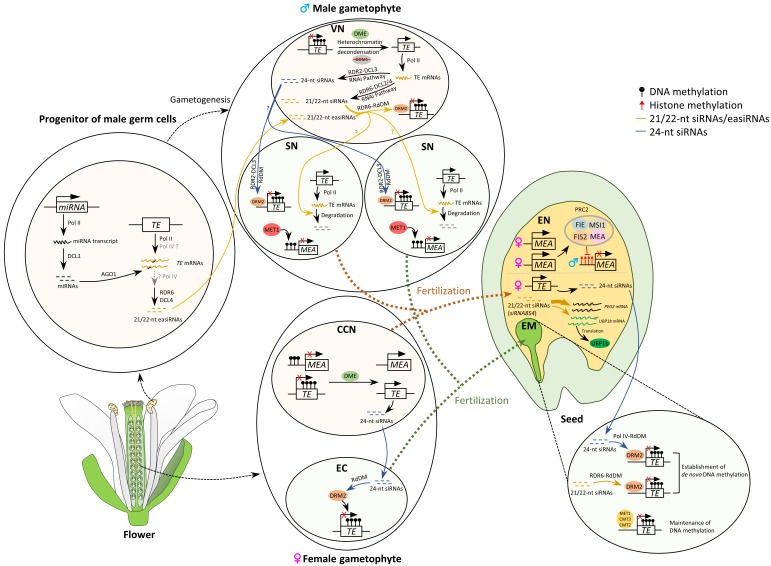
A simplified model of DNA methylation and siRNA reprogramming in gametes and during early embryogenesis in *Arabidopsis*. During gametogenesis, global DNA demethylation occurs in companion cells—vegetative and central cells [78,84,85,91]. DEMETER (DME) removes DNA methylation from imprinted genes and transposable elements (TEs) through the base excision repair pathway [94]. Expressed TEs due to demethylation and heterochromatin decondensation produce 21–24 nt small RNAs in companion cells [93]. In the vegetative cell, a portion of 21–22 nt small interfering RNAs (siRNAs) is generated through the ARGONAUTE1 (AGO1)-AGO2-DICER-LIKE4 (DCL4) RNAi pathway [93], and 21–22 nt epigenetically activated small RNA (easiRNA) biogenesis occurs during meiosis or early during gametogenesis [74,75]. In a progenitor of male germ cells, microRNA (miRNA) targets TEs and triggers polymerase IV (Pol IV)-mediated 21–22 nt easiRNA production, and the process requires Pol IV, RNA DEPENDENT RNA POLYMERASE6 (RDR6), and DCL4 [74,75]. In the vegetative cell, both 21–22 nt siRNAs generated from the RNAi pathway and 21–22 nt easiRNAs can trigger TE methylation through the RDR6-RNA-directed DNA methylation (RdDM) pathway. These easiRNAs/siRNAs might move from the vegetative cell to sperm cells to cause TE mRNA degradation. On the other hand, 24 nt siRNAs generated by the canonical RDR2-DCL3 RNAi pathway due to TE demethylation by DME in the vegetative cell might also move to the sperm cell to trigger methylation in TE by the canonical RDR2-DCL3 RdDM pathway. In the sperm cells, gene methylation is maintained by DNA METHYLTRANSFERASE1 (MET1), and TE methylation at CG, CHG, and CHH sites is maintained by MET1, CHROMOMETHYLASE3 (CMT3), and DOMAINS REARRANGED METHYLTRANSFERASE2 (DRM2)/CMT2, respectively (MET1/CMT3/CMT2 were not shown). The 24 nt siRNAs generated from expressed TEs in the central cell can move to the egg cell, where the siRNAs help maintain hypermethylation and silencing of imprinted genes and TEs through the canonical Pol IV-RdDM pathway. After fertilization, the hypomethylated alleles of imprinted genes in the central cell are passed onto the endosperm, which causes differential DNA methylation between parental alleles. PcG PRC2 complex including maternal MEA inhibits paternal *MEA* expression. In the endosperm, the 21–22 nt siRNAs, such as *siRNA854*, target *PATERNALLY EXPRESSED GENE2* (*PEG2*) mRNA to suppress its translation, and target *OLIGOURIDYLATE BINDING PROTEIN1b* (*UBP1b*) to regulate UBP1b protein levels by translation inhibition [95]. In the embryo, the 24 nt siRNAs can establish and silence TEs through the canonical Pol IV-RdDM pathway; the 21–22-nt siRNAs retained before fertilization help establish DNA methylation in TEs through the non-canonical RDR6-RdDM pathway. TE methylation at CG, CHG, and CHH sites is maintained by MET1, CMT3, and CMT2, respectively. Abbreviations are as follows: vegetative nucleus (VN), sperm nucleus (SN), central cell nucleus (CCN), egg cell (EC), embryo (EM), endosperm (EN).

DNA methylation and demethylation can regulate expression of imprinted genes in *Arabidopsis*. *MEA* is imprinted and critical for seed development [57]. Expression of the maternal *MEA* allele is antagonistically controlled by MET1 and DME in the *Arabidopsis* central cell [53,87,88]. MET1 represses maternal *MEA* expression by adding the methyl group to cytosine residues in the maternal *MEA* promoter, whereas DME activates maternal *MEA* expression through DNA demethylation in the *MEA* promoter region (Figure 2). The antagonism between MET1 and DME also regulates other imprinted genes in the late endosperm development, such as *FLOWERING WAGENINGEN* (*FWA*), homeodomain leucine zipper transcription factor, and *FIS2* [54,89]. It has been reported that a 200 bp *MEA* cis-regulatory region termed the imprinting control region (ICR) is required and sufficient for *MEA* imprinted expression by analyzing expression of the transgene *pMEA:GUS*, encoding β-glucuronidase (GUS) with the *MEA* promoter [90]. The authors suggested that maternal expression of *MEA* is indirectly controlled by antagonism between MET1 and DME, and proposed a model that maternal *MEA* expression depends on a yet unidentified positive transcription activator that is a direct target of MET1 and DME antagonism [90]. In short, it is critical to establish different methylation profiles in the egg and central cell during female gametogenesis, which are fertilized by sperm cells and develop into embryo and endosperm during embryogenesis, respectively.

## 3. Dynamic DNA Methylation in the Vegetative and Sperm Cells of Male Gametophyte

Compared with the seven-cell eight-nucleus female gametophyte in *Arabidopsis*, there are only three cells and two cell types in the male gametophyte. Recent studies reveal that vegetative and sperm cells have different methylation profiles, although they are only two divisions apart from the same precursor cell, microspore. The sperm nuclei have a slightly higher CG and slightly lower CHG methylation than the vegetative nucleus, but CHH DNA methylation in sperm nuclei (1.6%–2.1%) is only about half of that in the vegetative nucleus (4.0%–5.4%) (Figure 1) [78,84]. 

One major function of DNA methylation is to silence TEs. Surprisingly, some TEs in pollen vegetative nucleus are actually reactivated [91]. This is likely because *DME* is expressed in the vegetative cell but silenced in the sperm cell of the male gametophyte in addition to downregulation of *DECREASE IN DNA METHYLATION1* (*DDM1*), a gene required for DNA methylation [92]. Examining *Athila*, a retrotransposon, showed that activation of the TEs in the vegetative cell results in accumulation of 21 nt siRNAs that are likely to be transported to the sperm nuclei (Figure 2) [91]. Recently, it has been further revealed that siRNAs generated from mRNA transcripts in the vegetative cell can move to sperm cells and silence TE reporters, revealing a potential mechanism in which reactivation of TEs in companion cells can regulate the TE activity in their associated gametes (Figure 2) [93]. Further evidence was gathered by sequencing the methylome of the sperm cell, vegetative cell, and their precursor [84]. The results showed that CG and CHG DNA methylation was stably retained whereas CHH methylation was lost in retrotransposons in the sperm cells but was later restored in the embryo after fertilization. It was hypothesized that loss of methylation in TEs in sperm cells would regain their methylation later in part by the maternal 24 nt siRNAs produced by the RdDM pathway (Figure 2) [84]. 

DNA demethylation in the male and female companion cells by an active DME-dependent mechanism occurs before these cells are differentiated. Ibarra and colleagues examined methylation in pollen from *DME*/*dme* plants and found that CG sites were hypomethylated in wild-type vegetative cells compared to somatic tissue, however, hypomethylation was abolished in the *dme* vegetative cell nuclei, suggesting that DME is required for demethylation in the vegetative cell (Figure 2) [78]. Loci that showed decreased CHH methylation in the *dme* sperm had an increased CG methylation in the *dme* vegetative cell. Because loss of DME affects methylation status of the vegetative cell, which in turns affects sperm cell methylation, the authors proposed that DME expression in the vegetative cell is necessary for full methylation of a certain number of sperm TEs (small, AT-rich, and nucleosome-depleted euchromatic), suggesting that DNA demethylation in the companion cell produces a potential signal in the form of sRNAs that help prevent TE activation in the gametes [78].

It has been recently shown that the paternal TE-derived 21–22 nt sRNAs termed epigenetically activated small RNAs (easiRNAs) increased in the crossed seed in response to paternally increased ploidy, and that mutations from the paternal Pol IV suppressed easiRNA production and generated viable triploid seeds by inducing methylation in TEs through RdDM [75]. It has been further shown that the 21–22 nt easiRNA accumulation through Pol IV resulted from a highly conserved miRNA in plants, miR845, and targeting tRNA^Met^ primer-binding site (PBS) in long terminal repeats (LTR) [74]. These studies suggest that miRNAs produced in pollen affect siRNA accumulation, which can serve as a quantitative signal to balance parental dosage and affect seed development after fertilization.

## 4. Epigenetic Changes Regulate Early Embryogenesis in Plants

During early embryogenesis, body axes and major tissue layers are established. Although many factors involved in basic body plan establishment have been identified [96], underlying epigenetic mechanisms that potentially regulate cell differentiation in early embryogenesis have not been elucidated in many species. Patterns of DNA methylation during embryogenesis have been studied in many species, such as *Arabidopsis* (Figure 1), maize, and soybean [78,86,97]. DNA methylation can vary significantly at different developmental stages, especially during reproduction (Figure 1). DNA methylation in CG, CHG, and CHH contexts in the endosperm is lower than that in the embryo for each context, with an especially significant hypomethylation in CHH methylation (Figure 1) [78]. These dynamic patterns of DNA methylation appear to be important for successful reproduction, as abnormal DNA methylation affects normal planes and numbers of cell division and auxin hormone gradients in early embryogenesis in *Arabidopsis* [76].

Recently, multiple studies have clearly demonstrated that dynamic DNA changes occur during seed development [86,98,99,100,101]. Lin et al. profiled DNA methylome of *Arabidopsis* and soybean seeds at globular and cotyledon stages and in different seed regions, sub-regions, and tissues, and found that DNA methylation in the CHH context increases significantly during early seed development [86]. Analyzing the transcriptome and seed development of the quadruple *drm1 drm2 cmt2 cmt3* (*ddcc*) mutant that lacks CHH and CHG methylation, the authors thought that CHH and CHG methylation might not play an important role in seed development [86]. The above result is not a surprise because no obvious morphological phenotype has been reported about the *ddcc* mutant [102]. However, we cannot conclude that methylation is not involved in seed development because CHH and CHG methylation is necessary for silencing of more than 100 TEs during early seed development [86]. Moreover, double mutants for the maize *CMT3* and *DDM1* orthologs are embryonic lethal [103]. More importantly, DNA methyltransferase MET1 regulates early seed development including pattern formation of early cell division [76]. Furthermore, a recent study demonstrated that Pol IV is important for seed development in *Brassica rapa* [104]. Mutations in Pol IV-mediated small RNA pathway caused defects in seed development in *Brassica rapa*, suggesting Pol IV-mediated RdDM is involved in seed development. When the maternal parent is the mutant of *NUCLEAR RNA POLYMERASE D1* (*nrpd1*), a high percentage of seed abortion occurs. The self-pollinated *nrpd1* has reduced seeds per silique and small seeds with irregular shape [104]. The authors further showed that RdDM is critical in maternal sporophytic tissues, not in the female gametophyte or zygote [104]. Thus, these results reveal that DNA methylation is crucial for regulating transcriptional activation of TEs [105], gene expression, and early seed development, although some species may not show any morphological phenotypes with loss of methylation. 

## 5. Epigenetics Affects Late Embryogenesis, Seed Maturation, and Germination in Plants

Dynamic epigenetic changes occur during late embryogenesis and seed maturation and play a critical role in seed dormancy [86,98,99,100,101]. By using *Arabidopsis* as a model, DNA methylation dynamics during late seed development and germination have been revealed [86,98,99,100]. Although CG methylation does not show a significant change, CHH methylation increases significantly during embryogenesis and reaches the highest levels for most methylated sites in mature embryos [98,99]. Through whole-genome bisulfite sequencing, it has also been shown that methylation goes through dynamic changes during the process of seed maturation in soybean [86,101]. CHH methylation levels in soybean cotyledons changed from 6% at the early stage S2 to 10% and 11% at the late stages S6 and S8, respectively. Furthermore, genes with negative correlation between their promoter methylation and gene expression during soybean seed maturation were identified: 36 genes that have differentially methylated regions (DMRs) in the CG sites, 66 genes in the CHG sites, and 2136 genes in the CHH sites [101]. Profiling methylomes revealed that CHH methylation increases significantly in both soybean and *Arabidopsis* during seed development, but drops precipitously within the germinating seedling [86,98,99,100]. By comparing the methylation levels of TEs in germinating seeds and seedlings of wild type and *ros1 dml2 dml3* (*rdd*) triple demethylase mutant, no significant difference was detected, suggesting that active demethylation might not play a major role in this process [99]. It is likely that this global demethylation is due to methylation dilution caused by rapid cell divisions. Relatively stable CG and CHG methylation is likely maintained by active MET1 and CMT3, whereas dynamic CHH methylation is not fully established or maintained through RdDM and CMT2 pathways during seed germination [86,98,99,100].

Epigenetic and genetic pathways can crosstalk during seed development. *LEAFY COTYLEDON1* (*LEC1*) encodes a Heme Activator Protein (HAP3) subunit of the CCAAT binding factor and is required for cotyledon identity and embryo maturation [106,107]. *LEC1* is only expressed in the embryo and endosperm. A recent study has shown that *LEC1*, as a master regulator of seed development, not only directly controls a distinct set of genes during seed development [7], but also helps to reverse a silent chromatin at the *FLOWER LOCUS C* (*FLC*) locus to an active chromatin state and activates its expression de novo during early embryogenesis from the gametes. Importantly, this active chromatin is transmitted from the embryonic stage to postembryonic stages [108], an example of epigenetic memory, thus suggesting that epigenetic status during embryogenesis can be transmitted to an adult life. Epigenetic reprogramming of the vernalized state has shown that EARLY FLOWERING6 (EFL6) has histone H3 lysine 27 trimethylation (H3K27me3) demethylase activity [109]. Mutations in *EFL6* decrease its H3K27me3 demethylase activity in the reproductive tissues of adult plants, leaving H3K27me3 methylation at the *FLC* locus at higher levels and its expression at lower levels, and thus the epigenetic state of H3K27me3 at *FLC* in the reproductive tissues is maintained or passed onto the embryo of next generation [109]. These results suggest that an epigenetic reprogramming exists during plant reproduction.

## 6. PcG Proteins Regulate Seed Development

PcG proteins are critical for seed development. PcG proteins were identified and shown to function in seed development by genetic screening in *Arabidopsis*, and they include FIE, FIS2, MEA, and MSI1 [28,29,30,31]. Mutations in these PcG genes can bypass fertilization and initiate seed development in the absence of fertilization. The *fis*-class mutants (*fie*, *fis2*, *mea*, and *msi1*) show maternal gametophytic defects—seeds abort when the mutant allele is maternally inherited regardless of the paternal allele, and the mutants have a similar phenotype of an excessive endosperm proliferation resulting in early seed developmental arrest. However, obtaining the homozygous *fie* mutant plant from the cross of the maternal *fie* heterozygote with the paternal *cdka;1 fie* double heterozygote indicates that FIE is not essential for embryogenesis (CDKA: a cyclin-dependent kinase, a homologue of cdc2; the *cdka;1* mutant generates bicellular pollen grains, consisting of a vegetative and a single sperm-like cell), but FIE functioning in the PRC2 complex plays an essential role in catalyzing H3K27me3 in the genome [110].

FIS PRC2 complexes can target genes encoding MADS-box proteins in plants [111]. Expression of *PHERES1* (*PHE1*), a type I MADS-box gene, is regulated by the FIS2 PRC2 complex including MEA, FIE, and FIS2. Köhler et al. demonstrated that *PHE1* was not expressed before fertilization, and was transiently expressed in the embryo and the endosperm. The FIS-class proteins are necessary for silencing the maternal *PHE1* allele after fertilization. *PHE1* expression in the *fie* and *mea* mutants was about 5–10 fold higher than wild type and remained at high levels until 4 days after pollination (DAP), whereas *PHE1* expression levels decreased after 3 DAP in wild type. A decrease of *PHE1* expression in the *mea* mutant seed partially rescues the *mea* seed-abortion phenotype, suggesting that *PHE1* plays an important role in regulating *MEA* expression and is partially responsible for the lethal phenotype of *fis*-class mutants [111]. *AGAMOUS-LIKE62* (*AGL62*), a type I MADS-box gene, is another example demonstrating that PcG complexes regulate MADS domain proteins [112,113]. *AGL62* is specifically expressed in the *Arabidopsis* endosperm. During endosperm development, expression of *AGL62* is high during the syncytial phase and then decreases dramatically before cellularization. Kang et al. showed *AGL62* promotes nuclear proliferation and suppresses endosperm cellularization. The results suggest that the FIS-PRC2 complex is involved in silencing *AGL62* at the late stage of endosperm development [113].

Increasing maternal genome dosage can bypass genes essential for imprinting control [114], whereas eliminating the paternal contribution has a similar effect as increasing maternal genome dosage [115]. Although *FIS*-class genes are required for endosperm development, increasing ratio of maternal-to-paternal genome in the endosperm could allow normal seed development in the *fis* mutant [114]. This suggests that one of main functions of FIS-PRC2 is to balance the ratio of parental genome in seeds of flowering plants and reduce parental dosage conflict. This study also found that *AGL62* can reduce seed size in plants that have increased maternal genome dosage [114], presumably due to *AGL62* involvement in controlling the timing of endosperm cellularization. Recently, the FIS-PRC2 complex has been found to regulate the type I MADS-box genes, including many *AGL* genes [112]. Interestingly, the data suggest that the FIS-PRC2 complex might play dual roles in regulating type I MADS-box genes—a general role in suppressing gene expression at both maternal and paternal alleles during endosperm cellularization and a specialized role in silencing the maternal allele of imprinted genes [112]. It has been shown that the *FIS* genes function to balance the contribution of the paternal genome. When the paternal parent is the *cdka;1* mutant, the requirement of the maternal *MEA* and *FIE* alleles can be dispensable or the imprinted expression of *MEA* and *FIE* in developing seeds can be bypassed and viable homozygous *mea* or *fie* mutant seeds are developed [110,115]. This research showed that at certain conditions (i.e., in absence of the paternal contribution to the endosperm) the requirement of *FIS* genes in normal seed development can be dispensable [115]. Interestingly, CURLY LEAF (CLF), a H3K27me3 methyltransferase, has been shown to affect seed size [116]. The *clf-28* mutant seed is larger, heavier, and contains higher oil content. Furthermore, transcriptomic analysis shows that expression of a set of genes is repressed by *CLF*, and 46% of *CLF*-repressed genes are associated with epigenetic modification of H3K27me3 [116].

Decreased expression of PcG gene *OsFIE2* led to smaller seed in knockdown *OsFIE2* rice lines, and enzymes involved in storage proteins and starch synthesis were reduced [117]. In a related study, analysis of rice plants with developing seeds under moderate (34 °C) and high (42 °C) heat stress showed that moderate heat stress caused increased endosperm cellularization but high heat stress caused failure of cellularization [117]. Endosperm cellularization in rice was regulated by *OsFIE1*, which was susceptible to temperature alteration and its expression was anticorrelated with heat stress. Transgenic plants overexpressing *OsFIE1* had reduced seed size and premature cellularization. Furthermore, DNA methylation and histone modification were also altered in heat stress. This suggests that epigenetic regulation of endosperm development and thermal sensitivity of seed size could be linked through the PRC2 complex [118]. 

## 7. Effects of Histone Modifications on Seed Development

Histone modifications are involved in seed development. The *PcG* protein MEA is a histone H3 methyltransferase that methylates lysine 27 of histone H3 [119]. As described before, *MEA* is imprinted and regulates endosperm proliferation in seed development. The maternal *MEA* allele is expressed and antagonistically regulated directly or indirectly by MET1 and DME. The paternal *MEA* allele is silenced by DNA methylation and the silencing is maintained by the maternal FIS PRC2 (Figure 2). The maternal *MEA* allele expression silences the paternal *MEA* allele and also represses the transcription of imprinted genes such as *PHE1* that are paternally active and maternally silenced [53,120,121]. MEA suppresses expression of its target genes by H3K27me3 trimethylation, a repressive histone modification [119], that is associated with DMRs of the maternal alleles of paternally expressed genes (*PEGs*) in *Arabidopsis*, rice, and maize [97,122,123]. 

Histone H1 is classically viewed to compact chromatin into a higher order structure [124]. Recent evidence has showed that histone H1 variants have complex functions in chromatin remodeling and epigenetic regulation of plant growth and reproduction [125]. Epigenetically, mutations in H1 can cause DNA hypomethylation and hypermethylation in different genomic loci and result in misregulation of gene expression [126]. Histone H1 and DME interact, and H1 is involved in DME-mediated DNA demethylation in the endosperm [127]. Genetic analysis of the histone *h1* mutant indicated that the maternal histone *H1* allele is involved in imprinted expression of *MEA*, *FWA*, and *FIS2* in the endosperm of *Arabidopsis* [127]. The *H1* mutations cause increased methylation in the promoter of the maternal *MEA* and *FWA* alleles in the endosperm [127]. Interestingly, it has been found that DNA sequences in the heterochromatic regions require DDM1 and CMT2 for methylation, and that this process involves histone H1 in seedlings [20]. Furthermore, it has been shown that DDM1 allows nucleosome-bound DNA methylation and the double mutant *ddm1 h1* displays the strong linker-specific DNA methylation pattern in both *Arabidopsis* and mice [128]. These results suggest that naked DNA without nucleosomes is a preferred target of DNA methylation [128]. Recently, the STRUCTURE SPECIFIC RECOGNITION PROTEIN 1 (SSRP1), a subunit of the chromatin remodeler FACT (facilitates chromatin transactions), has been shown to co-localize with nuclear DME in vivo [129]. Furthermore, H1 mediates the requirement for FACT at some DME-target genes [129]. Interestingly, H1 is naturally depleted in the vegetative cell in pollen, and facilitates DME to access some heterochromatic TEs, demethylating and activating those TEs [130]. In summary, it seems that histone H1 variants might have subtly different roles in different tissues and cells at different developmental stages; for example, the egg cell versus the vegetative cells in regulating chromatin structure, DNA methylation, and expression of genes and TEs.

Histone H3 variants have been reported to participate in epigenetic reprogramming in plant reproduction. Although more than a dozen H3 variants are expressed in somatic cells, only a few H3 variants exist in male and female gametes in *Arabidopsis* [131]. After fertilization, H3 variants from male and female gametes are actively removed from the zygotic chromatin, and replaced with new somatic H3 variants in the embryo by de novo synthesis of H3 variants [131], suggesting reprogramming H3 variants in the zygote limit the inheritance of epigenetic information from parental genomes.

Histone acetylation is a vital chromatin modification for regulating gene expression during plant development [132]. Human SILENT INFORMATION REGULATOR6 (SIRT6) has been shown to deacetylate H3K9 and H3K56. *Oryza sativa* SIRTUIN1 (OsSRT1) is a histone deacetylase, closely related to the human SIRT6. Down-regulation of *OsSRT1*-induced expression of *RICE STARCH REGULATOR1* (*RSR1*) and amylase genes in developing seeds, which resulted in decreased starch synthesis, increased starch degradation, and abnormal seed development [133]. OsSRT1 was required for reducing histone H3K9 acetylation on genes and transposons during starch metabolism in developing seeds [133]. These results indicate that seed development is regulated, in part, by *OsSRT1*-mediated histone deacetylation.

*AtHDA7*, an *Arabidopsis HISTONE DEACETYLASE* (*HDAC*), is required for the female gametophyte development and embryogenesis in *Arabidopsis* [134]. Knocking out *AtHDA7* was shown to cause degeneration of micropylar nuclei at the four-nucleate embryo sac stage and delayed embryo development. Mutations in *AtHDA7* induced histone hyperacetylation, and significantly increased the transcription of other *HDACs* including *AtHDA6*. In addition, loss of *AtHDA7* expression affected expression of *ARABIDOPSIS HOMOLOG OF SEPARASE* (*AESP*), which was involved in female gametophyte and embryo development [135]. This suggests that maintaining appropriate histone acetylation patterns is necessary for plant reproductive development.

Studying MEIOTIC CONTROL OF CROSSOVERS1 (MCC1), a GENERAL CONTROL NON-REPRESSED PROTEIN 5 (GCN5)-related histone N-acetyltransferase, shows that histone hyperacetylation is important for meiosis in plants [136]. The *MCC1* gene was needed for meiosis. The *mcc1* siliques had 68% fewer seeds per silique than wild type and the embryo sacs failed to differentiate in 50% of *mcc1* ovules [136]. The *mcc1* mutant had defects in meiosis due to the *mcc1* pollen mother cells showing 30% increase in histone H3 acetylation [136]. Interestingly, a recent study showed histone acetyltransferase GCN5 also affected the fatty acid composition in *Arabidopsis* seeds. This suggests that histone acetylation affects not only ovule development but also fatty acid biosynthesis during late stage of seed maturation.

## 8. Small RNA Regulation is Critical in Seed Development

sRNAs are important regulators during plant growth and development [60], and especially important for reproductive development [137]. The homozygous male gametophytic *kokopelli* (*kpl*) mutant shows a high proportion of single fertilization event and 70% reduced seed set due to undeveloped ovules and aborted seeds [64]. *ARIADNE14* (*ARI14*), encoding a putative ubiquitin E3 ligase, and *KPL* generate sperm-specific natural cis-antisense siRNAs (cis-nat-siRNAs). In the *kpl* mutant, *ARI14* transcripts increased in sperm and fertilization was impaired. Furthermore, expression of *ARI14* was increased in the siRNA biogenesis mutants *dcl1*, *hen1*, *hyl1*, and *rdr2*, suggesting that the siRNA pathway is involved in generating cis-nat-siRNAs from the *KPL* locus [64].

The 21 nt and 24 nt phased siRNAs (phasiRNAs) are generated by RDR6 and DCL4/DCL5 [138,139,140]. Although premeiotic 21 nt phasiRNAs lack known targets, loss of MEIOSIS ARRESTED AT LEPTOTENE 1 (MEL1), an AGO protein that interacts with 21 nt phasiRNAs, results in abnormalities in tapetum formation and early arrest in pollen mother cells in rice [141,142]. The closest homolog of MEL1 in maize is AGO5, and the mutant *ago5-4* plants show defects in initiation of megagametogenesis and are unfertile in females. AGO4, a component involved in RdDM, is also involved in seed development. The *ago4-1* mutant has been shown to have reduced fertility, which was only associated with floral organ defects [143]. However, pollen grain viability varies in the mutant, suggesting that the RdDM pathway can be important for meiosis [144]. Interestingly, Walker et al. showed that RdDM affected meiosis in the male meiocyte [145]. They found hypermethylated loci in the male sexual lineage called sexual-lineage-hypermethylated loci (*SLHs*), which were caused by RdDM. Loss of methylation at the sexual-lineage-specific methylated (*SLM*) loci by interrupting RdDM affected splicing of *MULTIPOLAR SPINDLE1* (*MPS1*), a gene required for meiosis in *Arabidopsis*. Retention of the last intron of *MPS1* produces aberrant MPS1 protein that interferes with meiosis, resulting in abnormal meiosis and impaired tetrad formation [145].

siRNAs also provide important insights into the mechanism of parental genome dosage imbalance in seeds [146]. Seed size is affected by maternal and paternal genome ratio. Paternal-excess crosses (2n × 4n) delay endosperm cellularization and generate larger seeds, whereas maternal-excess crosses (4n × 2n) promote early cellularization and generate smaller seeds [147]. Although a mechanism to explain an imbalance of parental genome dosage could involve parent-of-origin-specific factors and imprinted genes, an alternative explanation could involve 24 nt siRNAs, which are also called p4-siRNAs, as RNA Pol IV is responsible for generating these RNAs [148,149,150]. Genetic analyses of reciprocal crosses between 2n and 4n showed that p4-siRNA production depends on the maternal genome dosage [146]. In the maternal-excess endosperm, maternal p4-siRNA levels increased and p4-siRNAs-associated *AGL* genes were repressed, causing early cellularization and smaller seeds. In the paternal-excess endosperm, there was a lower amount of maternal p4-siRNAs and upregulation of *AGLs*, resulting in nuclear proliferation and larger seeds [146]. There are inconsistent results as to whether p4-siRNAs are from the maternal [148] or from both maternal and paternal genomes [151,152]. Genome-wide sRNAs in whole seeds of three different *Arabidopsis* strains and their reciprocal crosses were analyzed and genes involved in RdDM were found to be expressed in both endosperm and embryo at 6 DAP, suggesting that both endosperm and embryo can potentially produce sRNAs [153]. On the contrary, 6%–24% sRNAs were from the paternal genome, which was similar to the fraction of the paternal mRNA reads in seeds. Furthermore, *NRPD1a* is primarily paternally expressed in all the reciprocal crosses [153]. These studies suggest that siRNAs produced by Pol IV are involved in regulating seed size mediated by RdDM.

Interestingly, the reciprocal crosses between *Arabidopsis thaliana* and *Arabidopsis lyrata* revealed that allelic dosage is regulated by a sRNA pathway in the endosperm [154]. The ratio between maternal and paternal transcripts is actively maintained by the Pol IV-mediated pathway. Mutations in Pol IV suppress negative effects of extra paternal genome dosage. Inheritance of a paternal Pol IV mutation can rescue the seed abortion phenotype caused by excess paternal genome dosage [154]. In a following up study, RdDM activity from the paternal parent was shown to be sufficient to determine seed viability in the cross with paternal excess in *Arabidopsis* [155]. Misregulation in TEs and imprinted genes was found to be unlikely to cause seed abortion in the cross with paternal excess, and a model of a transcriptional buffering system of balanced gene expression between parental alleles was proposed [155]. The above results were not consistent with the role of easiRNAs in dosage effects on seed development discussed in the previous paragraph. As discussed before, the recent research shows that production of paternal easiRNAs regulates parental genome dosage [74,75]. Martinez et al. showed that Pol IV produced TE-derived 21–22 nt easiRNAs in pollen and loss of paternal Pol IV suppressed easiRNA biogenesis and restored CHH methylation of TEs mediated by RdDM [75]. One big difference was that they showed that these 21–22 nt easiRNAs depend on *Pol IV-DCL2/4-RDR6* non-canonical RdDM pathway, whereas the paternal mutations in *DCL3*, *RDR2*, and *Pol V* cannot rescue the seed abortion caused by paternal excess [74,75]. However, Satyaki and Gehring showed that mutations in canonical RdDM pathway genes (*Pol V* and *DRM2*) are sufficient for suppressing seed abortion with paternal excess [155]. One of the reasons to explain this discrepancy is that Martinez et al. used the *omission of second division* (*osd1*) mutant to generate conditions of paternal excess [75], whereas the other study used the true tetraploid wild type and mutants generated by treatment with colchicine [155]. One explanation is that different paternal parents were used in these two studies. Furthermore, seed viability is a combinational consequence of genetic/epigenetic status of seeds, and physiological and environmental interaction. Nevertheless, considering the fact that these studies all show that the paternal mutation of Pol IV can suppress seed abortion of paternal excess, it is quite puzzling that contradictory results were obtained.

## 9. Summary and Discussion

Seed development is controlled by programmed gene expression. To date, many genes have been identified and characterized as being important for seed development [156]. The research in the last decade has shown epigenetics is important for seed development, but it remains largely unclear how different epigenetic mechanisms interact to regulate seed development. Since the first report that DNA methylation regulates embryogenesis and seed development in plants [76,157], we now know much more about the role of DNA methylation, histone modifications, and sRNAs in regulating seed development. Furthermore, DNA methylomes of gametes, embryo, endosperm, and seeds at different developmental stages have been sequenced in *Arabidopsis* and other organisms, which has greatly increased our understanding of how epigenetics affects reproductive development [158]. Most results discussed in this review have been obtained from studying *Arabidopsis thaliana*, which is very resilient to epigenetic perturbations, differing it from other plant species or mammals. These studies have shown a significant variation of DNA methylation among gametes, different seed compartments, and seeds at different stages (Figure 1). It is not clear what exact mechanisms cause dynamic DNA methylation during reproduction and how it evolved.

DNA methylation occurs in CG, CHG, CHH contexts of genes, repeats, and TEs, and its levels are quite diverse. In flowering plants, one of the major functions of DNA methylation is to silence TEs and to maintain genome stability during plant growth and development, including gametogenesis and seed development. In addition to silencing TEs and repeats, DNA methylation, as an epigenetic modification, regulates gene expression during seed development. Low levels of methylation in the gene promoter have been shown to correlate with high expression for some genes in *Arabidopsis*, rice, and soybean [65,85,101]. Although DNA methylome has been sequenced in many plant species, it remains to be determined how many genes are directly regulated by DNA methylation during seed development.

Besides the general role in gene regulation, DNA methylation has been demonstrated to regulate expression of a specific set of genes, including PcG and imprinted genes in seed development. PcG genes *MEA* and *FIS2* are imprinted, and their maternal alleles are antagonistically regulated by MET1 and DME in the endosperm. The paternal *MEA* allele is silenced by DNA methylation and this silenced state is maintained by its maternal FIS PRC2 complex in the endosperm. To date, many genes have been revealed to show imprinted expression [46,51,55,57,58], and it is very likely that they are regulated by methylation and other epigenetic mechanisms. It remains to be experimentally proven how many genes are actually imprinted and their specific function in seed development [46,58]. 

DNA methylation reprogramming occurs in gametogenesis prior to fertilization and embryogenesis. In the endosperm, hypomethylated maternal alleles can cause expression of 24 nt sRNA that might move to the embryo to methylate the homologous genes and/or TEs through RdDM, fortifying the silencing of TEs in the embryo (Figure 2). Similarly, in the male gametophyte, DME demethylates CG sites of DME-targeted genes or nearby TEs. It is hypothesized that expressed siRNA from hypomethylated TEs in the vegetative nucleus can move to adjacent sperm cells to further silence TEs in the sperm cell that passes its genetic information to the zygote [78,84,91]. It seems that both canonical (*DCL3*, *RDR2*, *Pol IV*, and *Pol V*) and non-canonical (*DCL2/4*, *RDR6*, *Pol IV*, and *Pol II*) RdDM pathways exist during reproduction. Epigenetic reprogramming of methylation in female and male gametophytes and during seed development is to ensure overall genome stability and inheritance of traits to the next generation. It still needs to be experimentally demonstrated how sRNAs are moved from microspores and/or the companion cell to the gametes to silence targeted genes through the RdDM pathway. In addition, exact mechanisms of hybridization barriers between different ploidy parents remain elusive—it can be due to misregulation of TEs and imprinted genes by Pol IV-mediated sRNAs, transcriptional buffering of parental alleles [155], or subtle indirect effects of misregulation of genes and TEs caused by collective epigenetic modifications.

Histone modifications are critical for development. Multiple lines of evidence have shown that histone H3 methyltransferases (MEA), histone acetyltransferases (AtHDA7 and GCN5), and deacetylases (OsSRT1) are involved in regulating seed development. It is apparent that DNA methylation, histone modifications (methylation, acetylation, etc.), PcG proteins, parent-of-origin and parental dosage effects, RdDM, and dynamic sRNA homeostasis are, in part, an integral epigenetic system during seed development. It remains to be elucidated how these different epigenetic pathways intertwine to affect dynamic epigenetic status in sexual lineage, zygote, and embryogenesis, and maintain transgenerational epigenetic inheritance and variations in reproduction.
